# Feeling backwards? How temporal order in speech affects the time course of vocal emotion recognition

**DOI:** 10.3389/fpsyg.2013.00367

**Published:** 2013-06-24

**Authors:** Simon Rigoulot, Eugen Wassiliwizky, Marc D. Pell

**Affiliations:** ^1^Faculty of Medicine, School of Communication Sciences and Disorders, McGill UniversityMontreal, QC, Canada; ^2^McGill Centre for Research on Brain, Language and MusicMontreal, QC, Canada; ^3^Cluster of Excellence “Languages of Emotion”, Freie Universität BerlinBerlin, Germany

**Keywords:** vocal emotions, prosody, speech perception, auditory gating, acoustics

## Abstract

Recent studies suggest that the time course for recognizing vocal expressions of basic emotion in speech varies significantly by emotion type, implying that listeners uncover acoustic evidence about emotions at different rates in speech (e.g., *fear* is recognized most quickly whereas *happiness* and *disgust* are recognized relatively slowly; Pell and Kotz, 2011). To investigate whether vocal emotion recognition is largely dictated by the amount of time listeners are exposed to speech or the position of critical emotional cues in the utterance, 40 English participants judged the meaning of emotionally-inflected pseudo-utterances presented in a gating paradigm, where utterances were gated as a function of their syllable structure in segments of increasing duration from the *end* of the utterance (i.e., gated syllable-by-syllable from the *offset* rather than the onset of the stimulus). Accuracy for detecting six target emotions in each gate condition and the mean identification point for each emotion in milliseconds were analyzed and compared to results from Pell and Kotz (2011). We again found significant emotion-specific differences in the time needed to accurately recognize emotions from speech prosody, and new evidence that utterance-final syllables tended to facilitate listeners' accuracy in many conditions when compared to utterance-initial syllables. The time needed to recognize *fear*, *anger*, *sadness*, and *neutral* from speech cues was not influenced by how utterances were gated, although *happiness* and *disgust* were recognized significantly faster when listeners heard the end of utterances first. Our data provide new clues about the relative time course for recognizing vocally-expressed emotions within the 400–1200 ms time window, while highlighting that emotion recognition from prosody can be shaped by the temporal properties of speech.

## Introduction

Emotional events, and more specifically social displays of emotion—the expression of a face, the tone of a speaker's voice, and/or their body posture and movements—must be decoded successfully and *quickly* to avoid negative outcomes and to promote individual goals. Emotional expressions vary according to many factors, such as their mode of expression (auditory/visual), valence (positive/negative), power to arouse (low/high), antecedents, and potential outcomes (see Scherer, 2009 for a discussion). As early as the seventeenth century, these differences raised the question of the *specificity* of emotions; in his Traité *“Les Passions de l'Ame,”* the French philosopher Descartes proposed the existence of six “primary” emotions from which all other emotions are derived. In recent decades, studies demonstrating accurate pan-cultural recognition of emotional faces (Izard, 1971; Ekman, 1972) and distinct patterns of autonomic nervous system activity in response to certain emotions (e.g., Ekman et al., 1983; Levenson, 1992) have served to fuel the idea of a fixed set of discrete and hypothetically “basic” emotions, typically *anger, fear, disgust, sadness*, and *happiness*, although opinions vary (see Ekman, 1992; Sauter et al., 2010). Within this theoretical framework, expressions of basic emotion possess unique physical characteristics that render them discrete in communication when conveyed in the face as well as in the voice (Ekman, 1992), although the vast majority of this work has focused on communication in the facial channel.

The structure of *vocal* emotion expressions embedded in spoken language, or *emotional prosody*, is now being investigated systematically from different perspectives. Perceptual-acoustic studies show that basic emotions can be reliably identified and differentiated at high accuracy levels from prosodic cues alone, and that these expressions are marked by distinct acoustic patterns characterized by differences in perceived duration, speech rate, intensity, pitch register and variation, and other speech parameters (among many others, Cosmides, 1983; Scherer et al., 1991; Banse and Scherer, 1996; Sobin and Alpert, 1999; Johnstone and Scherer, 2000; Juslin and Laukka, 2003; Laukka et al., 2005; Pell et al., 2009). For example, speech rate tends to decrease when speakers are sad and increase when speakers experience fear; at the same time, differences in relative pitch height, variation, and other cue configurations serve to differentiate these (and other) emotional meanings (see Juslin and Laukka, 2003 for a comprehensive review). Similar to observations in the visual modality, cross-cultural studies on the identification of vocal emotions show that *anger*, *fear*, *sadness*, *happiness*, and *disgust* can be recognized by listeners at levels significantly above chance when they hear semantically-meaningless “pseudo-utterances” or utterances spoken in a foreign language (Scherer et al., 2001; Thompson and Balkwill, 2006; Pell et al., 2009; Sauter et al., 2010). These data argue that basic emotions conveyed by speech prosody exhibit a core set of unique physical/acoustic properties that are emotion-specific and seemingly shared across languages (Scherer et al., 2001; Pell et al., 2009).

A critical process that has been underestimated in the characterization of how vocal emotions are communicated is the *time course* for recognizing basic emotions in speech. In the visual modality, the time course for recognizing emotional facial expressions has been investigated by presenting static displays of facial expressions (Tracy and Robins, 2008) or animated face stimuli (Becker et al., 2012). In this latter study, the authors used a morphed continuum running from a neutral exemplar to either a happy or an angry expression and found that happy faces were recognized faster than angry faces, suggesting temporal specificities in the process for recognizing basic emotions in the visual modality (see Palermo and Coltheart, 2004). Since emotional meanings encoded by prosody can *only* be accessed from their temporal acoustic structure, it is surprising that comparative data on the time course for recognizing basic emotions from prosody remain sparse.

Recently, two studies (Cornew et al., 2010; Pell and Kotz, 2011) examined the temporal processing of vocal emotion expressions using a modified version of Grosjean's (1980) gating paradigm. The auditory gating procedure—originally designed to pinpoint how much acoustic information is needed for lexical access and word recognition—consists of artificially constructing “gates” as a function of specific time increments or of relevant linguistic units of spoken language; the gated stimuli are judged by listeners in blocks of increasing gate duration, typically starting at the onset of the relevant stimulus, where the last gate presented usually corresponds to the entire stimulus event (see Grosjean, 1996 for a discussion of methodological variables). An emotional variant of this paradigm considers how much acoustic information is needed for vocal emotions to be registered and consciously accessed for explicit recognition, using a forced-choice emotion-labeling paradigm. Given the hypothesis that acoustic patterns reflect “natural codes” that progressively activate stored conceptual information about basic emotions (e.g., Schirmer and Kotz, 2006; Wilson and Wharton, 2006), this emotional gating procedure allows inferences about the time course of emotion processing in the specific context of speech, and whether the time needed varies as a function of the emotional signal being transmitted.

In the first study, Cornew and colleagues (2010) presented English-like pseudo-utterances spoken in a *happy*, *angry*, or *neutral* prosody to English listeners spliced into 250 millisecond (ms) gates of increasing duration. Following each stimulus, participants made a three-choice forced response to identify the meaning conveyed. The authors found that listeners required less time (i.e., exposure to acoustic information) to identify *neutral* sentences when compared to *angry* and *happy* sentences, suggesting that vocal emotion expressions unfold at different rates (an effect the authors attributed to a *neutral* bias in perception). The idea that vocal emotions unfold at different rates was replicated by Pell and Kotz (2011), who gated English-like pseudo-utterances as a function of their *syllable structure* as opposed to specific time increments. Forty-eight English participants listened to 7-syllable utterances conveying one of five basic emotions (anger, disgust, fear, sadness, happiness) or neutral prosody, beginning with presentation of only the first syllable of the utterance, the first two syllables, and so forth until the full sentence was presented (a six-choice forced response was recorded). Emotion identification times were then calculated by converting the number of syllables needed to accurately identify the target emotion of each utterance without further changes in the participant's response at longer gate intervals, into their actual duration for recognition.

Results showed that there were important emotion-specific differences in the accuracy and time course for recognizing vocal emotions, with specific evidence that *fear*, *sadness*, *neutral*, and *anger* were recognized from significantly less acoustic information than *happiness* or *disgust*, from otherwise identical pseudo-utterances. Prosodic cues conveying *neutral*, *fear*, and *sadness* and *anger* could be detected from utterances lasting approximately 500–700 ms (*M* = 510, 517, 576, and 710 ms, respectively), whereas *happiness* (*M* = 977 ms) and *disgust* (*M* = 1486 ms) required substantially longer stimulus analysis. Despite the fact that Cornew et al. (2010) focused on a restricted set of emotions when compared to Pell and Kotz (3-choice vs. 6-choice task), and gated their stimuli in a different manner (250 ms increments vs. syllables), there were notable similarities between the two studies in the average times needed to identify neutral (444 vs. 510 ms), angry (723 vs. 710 ms), and happy expressions (802 vs. 977 ms, respectively), although Pell and Kotz's (2011) results show that this does not reflect a bias for recognizing neutral prosody as initially proposed (Cornew et al., 2010). Together, these studies establish that the time course of vocal emotion recognition in speech varies significantly according to the emotional meaning being conveyed, in line with results demonstrating emotion-specificity in facial emotion recognition (Becker et al., 2012), although the relative pattern of emotion-specific differences observed in the auditory vs. visual modality appears to be quite different as noted elsewhere in the literature using different experimental paradigms (e.g., Wallbott and Scherer, 1986; Paulmann and Pell, 2011).

Of interest here, closer inspection of Pell and Kotz's (2011) data reveal that recognition of *happiness* and *disgust*, in contrast to other basic emotions, improved at relatively long utterance durations (5–7 syllables); in fact, when full sentences were presented, recognition of *happy* prosody was comparable in accuracy to *sadness, anger*, and *fear* despite the fact that these latter emotions were recognized much more accurately than happiness following brief stimulus exposure. Some emotions such as *happiness* and *fear* seemed to be particularly salient when the last syllables were presented, leading to significant increases in recognition accuracy at the end of utterances in that study. These results imply that the amount of time needed to identify basic emotions from prosody depends partly on the *position* of salient acoustic properties in speech, at least for certain emotions. Interestingly, Pell (2001) reported that *happy* utterances exhibit unique acoustic differences in sentence-final position when compared to linguistically identical *angry*, *sad*, and *neutral* utterances, arguing that the position of acoustic cues, and not just time, is a key factor in communicating vocal emotions in speech. Other data underscore that the ability to recognize basic emotions varies significantly depending on the channel of expression—i.e., whether conveyed by facial expressions, vocal expressions, or linguistic content (Paulmann and Pell, 2011)—with evidence that *fear, sadness, anger*, and *neutral* are effectively conveyed by speech prosody, whereas other emotions such as *happiness* or *disgust* are much more salient in other channels (Paulmann and Pell, 2011). These findings raise the possibility that when basic emotions are preferentially communicated in channels other than the voice, vocal concomitants of these emotions are encoded and recognized somewhat differently; for example, they could be partly marked by local variations in acoustic cues that signal the interpersonal function or social relevance of these cues to the listener at the end of a discourse, similar to how the smile may reflect *happiness* or may serve social functions such as appeasement or dominance (Hess et al., 2002).

Further investigations are clearly needed to understand the time course of vocal emotion recognition in speech and to inform whether temporal specificities documented by initial studies (Cornew et al., 2010; Pell and Kotz, 2011) are solely dictated by the *amount of time* listeners require to identify vocal emotions, or whether linguistic structure plays a role for identifying some emotions. We tested this question using the same gating paradigm and emotionally-inflected utterances as Pell and Kotz (2011), although here we presented pseudo-utterances gated syllable-by-syllable from the *offset* rather than the onset of the stimulus (i.e., in a “backwards” or reverse direction) to test whether recognition times depend on how utterances are presented. If the critical factor for recognizing certain basic emotions in the voice is the unfolding of acoustic evidence over a set period of time, we expected similar outcomes/emotion identification times as those reported by Pell and Kotz (2011) irrespective of how utterances were gated; this result would establish that modal acoustic properties for understanding emotions tend to permeate the speech signal (perhaps due to their association with distinct physiological “push effects,” e.g., Scherer, 1986, 2009) and are decoded according to a standard time course. However, if important acoustic cues for recognizing vocal emotions are differentially encoded within an utterance, we should witness significantly different emotion identification times here when utterances are gated from their offset when compared to when they are presented from their onset (Pell and Kotz, 2011). This result could supply evidence that some emotions are “socialized” to a greater extent in the context of speech prosody through functionally distinct encoding processes.

## Methods

### Participants

Forty native English speakers recruited through campus advertisements (20 men/20 women, mean age: 25 ± 5 years) took part in the study. All participants were right-handed and reported normal hearing and normal or corrected-to-normal vision. Informed written consent was obtained from each participant prior to the study which was ethically approved by the Faculty of Medicine Institutional Review Board at McGill University (Montréal, Canada). Before the experiment, each participant completed a questionnaire to establish basic demographic information (age, education, language skills).

### Stimuli

As described by Pell and Kotz (2011), the stimuli were emotionally-inflected pseudo-utterances (e.g., *The placter jabored the tozz*) selected from an existing database of recorded exemplars, validated and successfully used in previous work (e.g., Pell et al., 2009; Paulmann and Pell, 2010; Rigoulot and Pell, 2012). Pseudo-utterances mimic the phonotactic and morpho-syntactic properties of the target language but lack meaningful lexical-semantic cues about emotion, allowing researchers to study the isolated effects of emotional prosody in speech (see Scherer et al., 1991; Pell and Baum, 1997 for earlier examples). The selected utterances were digitally recorded by two male and two female speakers in a sound-attenuated booth, saved as individual audio files, and perceptually validated by a group of 24 native listeners using a seven forced-choice emotion recognition task (see Pell et al., 2009, for full details). For this study we selected a subset of 120 pseudo-utterances that reliably conveyed *anger, disgust, fear, happiness, sadness* and *neutral* expressions to listeners (20 exemplars per emotion). Thirteen unique pseudo-utterance phrases produced by the four speakers to convey each emotion were repeated throughout the experiment (see Section Appendix). These sentences were the same in their (pseudo) linguistic content as those presented by Pell and Kotz (2011), although the precise recordings selected here were sometimes different because some phrases were emotionally expressed by a different speaker (75% of the chosen recordings were identical to those presented by Pell and Kotz, 2011). For all emotions, the target meaning encoded by prosody for these items was recognized at very high accuracy levels based on data from the validation study (anger = 86%; disgust = 76%; fear = 91%; happiness = 84%; sadness = 93%; neutral = 83%, where chance in the validation study was approximately 14%). Pseudo-utterances conveying each emotion were produced in equal numbers by two male and two female speakers and were all seven syllables in length prior to gate construction.

### Gate construction

Each utterance was deconstructed into seven gates according to the syllable structure of the sentence using Praat speech analysis software (Boersma and Weenink, 2012). As we were interested in the time course of emotion recognition when utterances were presented from their end to their beginning, the first Gate (Gate_7) of each stimulus consisted of only the last syllable of the utterance, the second gate (Gate_6-7) consisted of the last two syllables, and so on to Gate_1-7 (presentation of the full utterance). For each of the 120 items, this procedure produced seven gated stimuli (Gate_7, Gate_6-7, Gate_5-7, Gate_4-7, Gate_3-7, Gate_2-7, Gate_1-7) each composed of a different number of syllables (120 × 7 = 840 unique items). Note that since the onset of most gated stimuli occurred at a syllable break *within* the utterance (with the exception of Gate_1-7), these stimuli gave the impression of being “chopped off” at the beginning and starting abruptly. As shown in Table 1, the duration of items presented in each gate condition differed by emotion type due to well-documented temporal differences in the specification of vocal emotion expressions (Juslin and Laukka, 2003; Pell and Kotz, 2011).

**Table 1:** **Duration of the stimuli presented in the experiment in each gate duration condition as a function of emotion**.

	**Emotion**	**Gate condition (# syllables)**						
		**G_7**	**G_6-7**	**G_5-7**	**G_4-7**	**G_3-7**	**G_2-7**	**G_1-7**
Duration	Anger	370	585	771	1004	1230	1581	1759
	Disgust	481	748	984	1290	1555	1958	2153
	Fear	329	498	636	795	930	1151	1269
	Sadness	405	626	815	1071	1286	1645	1846
	Happiness	375	601	763	978	1164	1478	1648
	Neutral	354	540	703	896	1122	1401	1553

*Pseudo-utterances were always gated at syllable boundaries from the offset of the utterance in gates of increasing syllable duration*.

### Experimental design/procedure

Participants were invited to take part in a study of “communication and emotion”; they were seated in a quiet, dimly lit room at a 75 cm distance from a laptop screen. SuperLab 4.0 software (Cedrus, USA) was used to present auditory stimuli played over volume-adjustable, high-quality headphones.

Seven presentation blocks were built, each containing 120 items with the same gate duration (i.e., number of syllables) presented successively in blocks of increasing syllable duration. The first block contained all Gate_7 stimuli (tokens with only the last syllable), the second block contained all Gate_6-7 stimuli (last two syllables), and so on until the Gate_1-7 block containing the full utterances was presented. As in Pell and Kotz (2011), this block design was chosen to mitigate potential artifacts such as response perseveration (Grosjean, 1996). Individual stimuli were randomized within blocks, and participants were instructed to identify the emotion expressed by the speaker as accurately and quickly as possible from six alternatives presented on the computer screen (*anger, disgust, fear, sadness, happiness, neutral*). Responses were recorded by a mouse click on the corresponding emotion label. Following the emotion response, a new screen appeared asking participants to rate how confident they were about their emotional decision along a 7-point scale, where 1 indicated they were “very unsure” and 7 meant that they were “very sure” about their judgment. After recording the confidence rating, a gap of 2 s separated the onset of the next trial.

Participants completed ten practice trials at the beginning of the testing session and additional practice trials prior to each block to become familiar with stimuli representing each gate duration condition. Participants were allowed to adjust the volume during the first practice block of each session. Since the volume of our stimuli was homogenized, only one adjustment at the beginning was necessary to meet the participants' individual preferences. The full experiment was administered during two separate 60-min sessions (session 1 = first three gate conditions, session 2 = last four gate conditions) to reduce fatigue and familiarity with the stimuli. Participants received $25 CAD compensation for their involvement.

### Statistical analyses

Participants' ability to identify emotional target meanings (% correct) and their associated confidence ratings (7-pt scale) were each analyzed. From the uncorrected accuracy (hit) rates of each participant, Hu-scores were computed for each gate and emotion to adjust for individual response biases when several emotion categories are used (see Wagner, 1993). The computation of Hu-scores takes into account how many stimulus categories and answer possibilities are given in the forced choice task. If only two stimulus categories and two answer possibilities are used (e.g., neutral and anger) the Hu-score for the correct identification of one category, say anger, would be computed as follows: *H*u = *a*/*a* + *b* × *a*/*a* + *c*. Here *a* is the number of correctly identified stimuli (anger was recognized as anger), *b* is the number of misidentifications, in which anger was incorrectly labeled as neutral, whereas *c* is the number of misidentifications, in which neutral was incorrectly labeled as anger. Wagner (1993) describes the Hu-scores as “[…] the joint probability that a stimulus category is correctly identified given that it is presented at all and that a response is correctly used given that it is used at all.”

Hu-scores and confidence scores were submitted to separate 7 × 6 ANOVAs with repeated measures of gate duration (seven levels) and emotion (*anger, disgust, fear, happiness, sadness, neutral*). To infer how much time participants required to correctly identify emotions, we computed the “emotion identification point” for each of the 120 pseudo-utterances by determining the gate condition where a participant identified the target emotion without subsequent changes at longer gate durations of the same stimulus. The emotion identification points were then transformed into “emotion identification times” by converting the number of syllables needed to identify the target into the exact speech duration in milliseconds, which was then averaged across items for each participant (see Pell and Kotz, 2011 for detailed procedures). Of the 4800 possible identification points (20 items × 6 emotions × 40 participants), 419 items that were not correctly identified by a participant even when the full utterance was presented were labeled as “errors” and omitted from the calculation of emotion identification times (a total of 4381 data points were included). Mean emotion identification times were submitted to a one-way ANOVA with repeated measures on emotion (*anger, disgust, fear, happiness, sadness, neutral*).

Since the stimuli, procedures, and analyses adopted here were virtually identical to those of Pell and Kotz (2011), our experiment allows unprecedented comparisons of how recognition of emotional prosody evolves over time as a function of the gating *direction*, shedding light on how the position of acoustic patterns for detecting emotions influences recognition processes. For each of our three dependent measures (accuracy scores, confidence ratings, emotion identification times), we therefore performed a second analysis to directly compare the current results to those of Pell and Kotz (2011) by entering the between-groups factor of Presentation Direction (gating from offset vs. onset). Separate *t*-tests first compared the age and education (in years) of the current participant group (*n* = 40) with participants studied by Pell and Kotz (2011, *n* = 48); there was no difference in the formal education of the two samples [17 vs. 16 years, respectively; *t*_(86)_ = 1.548; *p* = 0.125], although participants in the present study were older on average [25 vs. 22 years; *t*_(86)_ = 2.578; *p* = 0.012]. Given the age difference, we entered age as a covariate in separate mixed ANCOVAs on the Hu-scores, confidence ratings, and emotion identification times as described above with the additional grouping variable of presentation Direction (onset, offset) of key theoretical interest in these analyses. For all statistical analyses, a significance level of 5% (two-sided) was selected and *post-hoc* comparisons (Tukey's HSD, *p* < 0.05) were applied whenever a significant main or interactive effect was observed.

## Results

### Accuracy (HU-scores) and confidence ratings

#### Effects of backwards gating on accuracy and confidence scores

**Figure 1:**
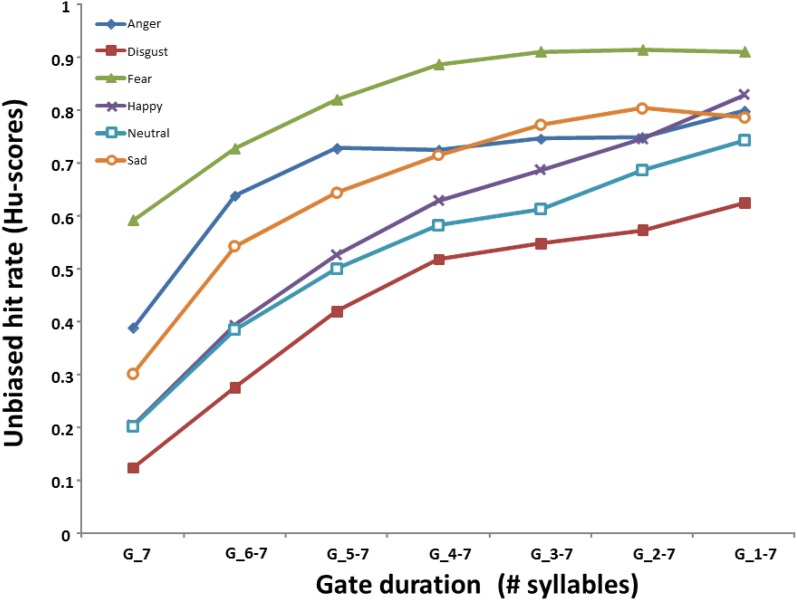
**Mean Hu-scores (unbiased accuracy) for each emotion as a function of the gate duration (number of syllables)**.

Table 2 shows the mean accuracy of participants (% correct target recognition) in each emotion and gate condition when utterances were presented from their offset, prior to correcting these scores for participant response bias. A 7 (Gate) × 6 (Emotion) ANOVA performed on the *unbiased* emotion recognition rates (i.e., calculated Hu-Scores) revealed a main effect of Gate duration [*F*_(6, 228)_ = 390.48; *p* < 0.001], Emotion [*F*_(5, 190)_ = 142.57; *p* < 0.001], and a significant interaction of these factors [*F*_(30, 1140)_ = 10.684; *p* < 0.001]. Post hoc (Tukey's) tests of the interaction first considered how the recognition of each emotion evolved as a function of gate duration when sentences were gated from their offset. As shown in Figure 1, the recognition of *fear*, *anger*, and *sadness* significantly improved over the course of hearing the first three gates (i.e., the last three syllables of the utterance, *p*s < 0.003) with no further accuracy gains by the fourth gate condition (Gate_4-7, *p*s > 0.115). In contrast, accurate recognition of *neutral*, *happiness*, and *disgust* each significantly improved over a longer time frame corresponding to the first four gate conditions (Gate_7 to Gate_4-7, *p*s < 0.001) without further changes after this point (*p*s > 0.087).

**Table 2:** **Mean accuracy (% target recognition) of the 40 listeners who judged pseudo-utterances conveying each emotion according to the gate duration, when utterances were gated from the offset of the sentence**.

	**Emotion**	**Gate condition (# syllables)**						
		**G_7**	**G_6-7**	**G_5-7**	**G_4-7**	**G_3-7**	**G_2-7**	**G_1-7**
Accuracy	Anger	51.9 (33.9)	73.0 (25.7)	79.9 (22.6)	79.0 (26.9)	80.3 (26.3)	81.8 (22.9)	85.8 (17.9)
	Disgust	27.5 (16.9)	44.3 (17.2)	59.3 (13.1)	64.3 (15.7)	71.0 (15.2)	71.4 (15.8)	74.5 (14.5)
	Fear	77.5 (15.6)	85.4 (16.0)	91.9 (8.3)	95.9 (3.7)	96.3 (4.1)	95.4 (4.7)	94.6 (3.9)
	Sadness	65.6 (22.8)	83.9 (13.9)	87.0 (11.3)	90.9 (12.1)	92.8 (7.5)	95.1 (5.0)	94.4 (6.4)
	Happiness	30.6 (27.9)	53.8 (34.4)	66.1 (32.7)	71.8 (32.0)	77.6 (25.9)	82.4 (24.5)	89.1 (13.5)
	Neutral	55.3 (12.8)	68.5 (11.8)	73.4 (12.5)	83.9 (10.8)	81.5 (11.2)	85.4 (8.5)	86.6 (8.2)

*Standard deviations are shown in parentheses*.

Further inspection of the interaction then looked at emotional differences on accuracy at each gate condition. When listeners heard only the utterance-final syllable (Gate_7), *fear* and *anger* prosody were recognized significantly better than all other emotional voices (*p*s < 0.006), and *fear* was also recognized significantly better than *anger* (*p* < 0.001). After fear and anger, *sad* expressions were identified significantly better from the last syllable than *happy* and *neutral* expressions (*p*s < 0.001), which did not differ (*p* = 1.000), followed by *disgust* which was recognized more poorly than any other emotion (*p*s < 0.046). This pattern was similar for stimuli composed of the last two and the last three syllables (Gate_6-7 and Gate_5-7, respectively) but changed somewhat as stimulus duration increased. After presenting the last four syllables (Gate_4-7), *fear* continued to exhibit the highest accuracy score (this was true in all gate conditions; *p*s < 0.017) but recognition of *anger* and *sad* expressions was equivalent (*p* = 1.0), followed by *happiness* which was recognized significantly better than *disgust* (*p* < 0.001). After the last five syllables were presented (Gate_3-7), *angry*, *sad* and *happy* sentences were recognized at a similar rate (*p*s > 0.555), surpassing *neutral* and *disgust* (*p*s < 0.001). In the two longest gate conditions (Gate_2-7, Gate_1-7), accuracy scores for *anger*, *sad*, *happy* and *neutral* sentences were not statistically different (*p*s > 0.407) while vocal expressions of *fear* and *disgust* were respectively the best and worst recognized from speech prosody (*p*s < 0.017).

The analysis of associated confidence ratings (on a scale of 1–7) was restricted to trials in which the emotional target of the prosody was correctly identified. Two male participants who failed to recognize any of the *disgust* expressions (producing an empty cell) were excluded from this analysis. The ANOVA on the confidence scores revealed a main effect of gate duration [*F*_(6, 192)_ = 48.653; *p* < 0.001], a main effect of emotional prosody [*F*_(5, 160)_ = 46.991; *p* < 0.001] and a significant interaction of Gate × Emotion [*F*_(30, 960)_ = 3.814; *p* < 0.001]. Confidence scores tended to increase with stimulus/gate duration, although there were differences across emotions as a function of gate duration. After listening to the final one or two syllables, participants were significantly more confident about their detection of *fear* and *anger* (*p*s < 0.001) and least confident when they correctly recognized *neutral* and *disgust* (*p*s < 0.001). Confidence ratings for *happiness* and *sadness* were between those extremes, differing significantly from the other two emotion sets (*p*s < 0.048). By the third gate condition (Gate_5-7), confidence about *neutral* prosody began to increase over *disgust* (*p* < 0.001), and by the fourth gate condition and when exposed to longer stimuli, confidence ratings for *fear*, *anger*, *happiness*, and *sadness* were all comparable, although confidence about *disgust* remained significantly lower even when full utterances were presented (Gate_1-7).

#### Impact of gating direction on accuracy and confidence scores

**Figure 2:**
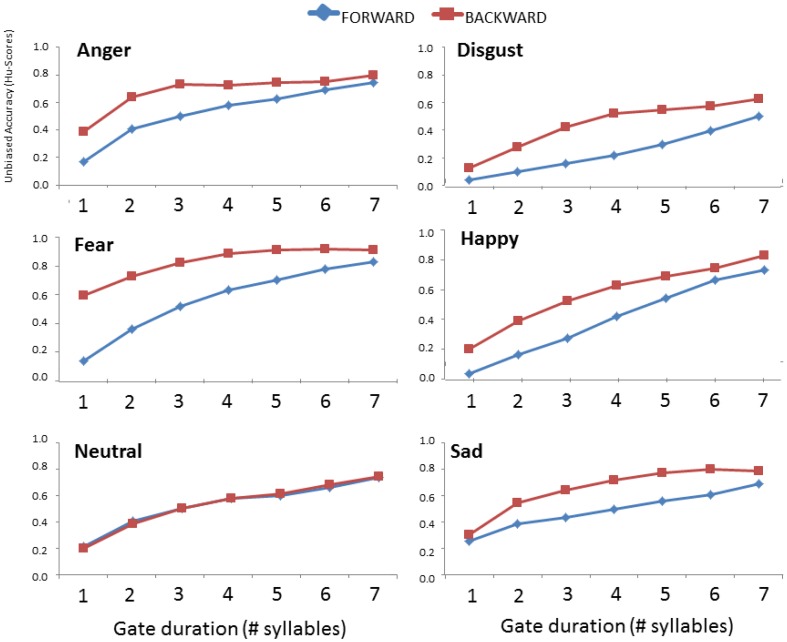
**Comparison of mean accuracy (Hu) scores for each emotion as a function of gate duration (number of syllables) and the direction of presentation (forward vs. backward)**. Data in the forward condition are taken from Pell and Kotz (2011).

The 2 × 7 × 6 ANCOVA on Hu-scores gathered here and by Pell and Kotz (2011) showed a significant three-way interaction of Direction, Gate duration, and Emotion [*F*_(30, 2550)_ = 12.636; *p* < 0.001]. This interaction allowed us to explore the influence of presentation direction (onset vs. offset) on the accuracy of emotional prosody recognition as additional syllables revealed acoustic evidence about each emotion; these relationships are demonstrated for each emotion in Figure 2. Step-down analyses (2x7 ANOVAs) showed that the interaction of Direction × Gate duration was significant for *anger* [*F*_(6, 516)_ = 14.218; *p* < 0.001], *fear* [*F*_(6, 516)_ = 33.096; *p* < 0.001], *disgust* [*F*_(6, 516)_ = 10.851; *p* < 0.001], *sadness* [*F*_(6, 516)_ = 11.846; *p* < 0.001], and *happiness* [*F*_(6, 516)_ = 9.663; *p* < 0.001]. For each of these emotions, recognition always improved when the *end* of utterances were heard first (i.e., when gated from their offset vs. onset), although the temporal region where accuracy improved within the utterance varied by emotion type. *Post-hoc* comparisons showed that *anger* and *fear* were recognized significantly better in the offset presentation condition even when little acoustic evidence was available; listeners detected *anger* better over the course of the first to third syllable in the offset vs. onset condition, and over the course of the first to sixth syllables for *fear* (*p*s < 0.001). *Happiness* showed an advantage in the offset condition beginning at the second up to the fourth gate (*p*s = 0.027), *disgust* showed a similar advantage beginning at the third to the fifth gate (*p* < 0.049), and *sadness* displayed the offset advantage beginning at the third up to the sixth gate (*p*s < 0.031). Interestingly, there was no effect of the direction of utterance presentation on the recognition of *neutral* prosody [*F*_(6, 516)_ = 0.409; *p* = 0.873].

**Figure 3:**
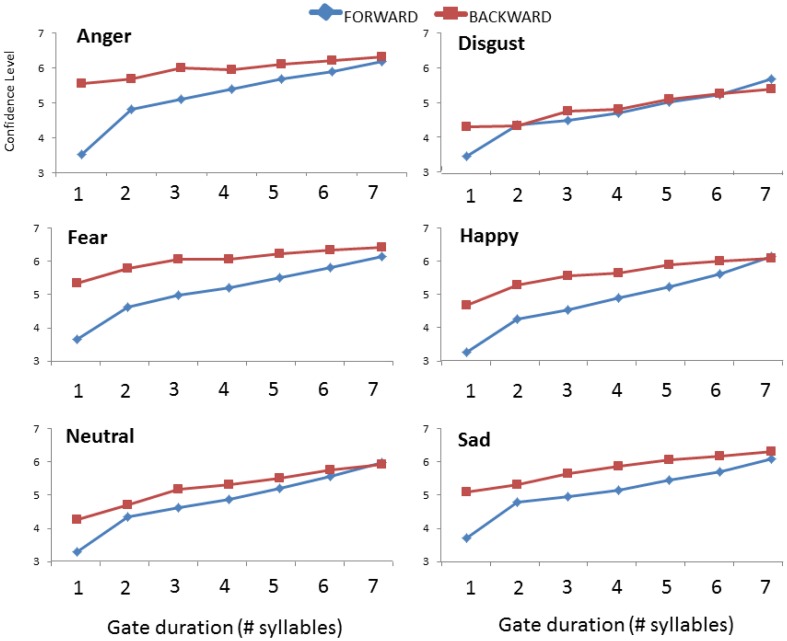
**Comparison of mean confidence ratings for each emotion as a function of gate duration (number of syllables) and the direction of presentation (forward vs. backward)**. Data in the forward condition are taken from Pell and Kotz (2011).

The ANCOVA on confidence ratings between studies yielded a significant three-way interaction of Direction, Gate duration and Emotion [*F*_(30, 2370)_ = 4.337; *p* < 0.001]. Step-down analyses (2 × 7 ANOVAs) run separately by emotion showed that the interaction of Direction × Gate duration was significant for *anger* [*F*_(6, 516)_ = 35.800; *p* < 0.001], *fear* [*F*_(6, 516)_ = 19.656; *p* < 0.001], *happiness* [*F*_(6, 504)_ = 18.783; *p* < 0.001], and *sadness* [*F*_(6, 516)_ = 10.898; *p* < 0.001]. Listeners were more confident that they had correctly identified these four emotions only when one syllable was presented in isolation (i.e., at the first gate duration, *ps* < 0.049), with increased confidence when they heard the sentence-final as opposed to the sentence-initial syllable. For *disgust* and *neutral*, the two-way interaction was also significant [*F*_(6, 492)_ = 7.522; *p* < 0.001; *F*_(6, 516)_ = 7.618; *p* < 0.001, respectively] but *post hoc* tests revealed only minor differences in the pattern of confidence ratings in each presentation condition with no differences in listener confidence at specific gates (*p*s > 0.618). These patterns are illustrated for each emotion in Figure 3.

### Emotion identification times

#### Effects of backwards gating on the time course of vocal emotion recognition

As described earlier, emotion identification times were computed by identifying the gate condition from sentence offset where the target emotion was correctly recognized for each item and participant, which was then converted into the precise time value of the gated syllables in milliseconds. A one-way ANOVA performed on the mean emotion identification times with repeated measures of emotion type (*anger, disgust, fear*, *happiness*, *sadness* and *neutral*) revealed a highly significant effect of emotion [*F*_(5, 190)_ = 113.68; *p* < 0.001]. As can be seen in Figure 3, *fearful* voices were correctly identified at the shortest presentation times (*M* = 427 ms), significantly faster than *sadness* (*M* = 612 ms), *neutral* (*M* = 654 ms) and *anger* (*M* = 672 ms) which did not significantly differ one from another. These emotions required significantly less time to identify than *happiness* (*M* = 811 ms), which in turn took significantly less time than *disgust* (*M* = 1197 ms) which required the longest stimulus exposure for accurate recognition (all *p*s < 0.001).

#### Impact of gating direction on emotion identification times

**Figure 4:**
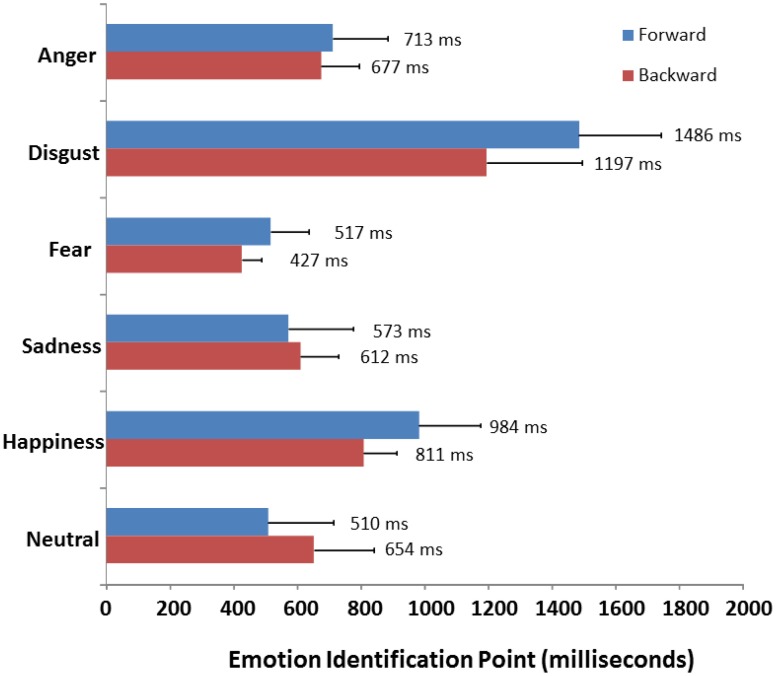
**Comparison of mean identification points (in milliseconds) for each emotion as a function of direction of presentation (forward vs. backward)**. Data in the forward condition are taken from Pell and Kotz (2011).

Finally, a 2 × 6 (Direction × Emotion) mixed ANCOVA was performed on the emotion identification times to compare the present results to those of Pell and Kotz (2011); this analysis revealed a significant interaction of presentation Direction and Emotion [*F*_(5, 425)_ = 13.235; *p* < 0.001] as also shown in Figure 4. The average time listeners required to correctly identify emotional prosody was significantly reduced when syllables were presented from the offset vs. onset of utterances, but only for *disgust* (*p* < 0.001) and *happiness* and (*p* = 0.050). In contrast to accuracy and confidence ratings, the manner in which utterances were gated had no significant impact on the amount of time listeners needed to recognize *fear*, *sadness*, *anger*, or *neutral* prosody (all *p*s > 0.157).

## Discussion

Following recent work (Cornew et al., 2010; Pell and Kotz, 2011), this experiment sought a clearer understanding of how vocal expressions of basic emotion reveal their meanings in speech using a modified version of the gating paradigm, where emotionally-inflected pseudo-utterances were truncated and presented in excerpts of increasing syllable duration from the *end* of an utterance. While the current manner for presenting our stimuli might bear no immediate resemblance to how emotional speech is encountered in structured conversations–especially because our stimuli were only auditory and not spontaneously produced (see Barkhuysen et al., 2010 for a discussion on this topic)—our performance measures may help to understand some processes involved when listeners “walk in” on an emotional conversation, or have their attention directed to emotional speech in the environment that is already in progress, an experience that is common to everyday life. Critically, our design allowed important hypotheses to be tested concerning the evolution and associated time course of emotional prosody recognition (in English) as listeners are progressively exposed to representative acoustic cue configurations. In line with past findings, we found that listeners tended to be most accurate at recognizing vocal expressions of *fear* (Levitt, 1964; Zuckerman et al., 1975; Paulmann and Pell, 2011; Pell and Kotz, 2011) and least accurate for *disgust* (e.g., Scherer et al., 1991; Banse and Scherer, 1996) irrespective of how many syllables/gates were presented. Expressions of *fear* were also recognized from the shortest stimulus duration, implying that listeners need minimal input to recognize this emotion in speech (Pell and Kotz, 2011). Interestingly, emotion identification times were significantly reduced for certain emotions (*happiness, disgust*) when sentences were presented from their offset rather than their onset, and there were other apparent “advantages” to recognizing emotion prosody when listeners were first exposed to the *end* of utterances. These effects and their implications are discussed in detail below.

### Effects of gating direction and cue location on vocal emotion recognition

Our data show that recognition of vocal emotions generally improves with the number of syllables presented, even when listeners hear utterance fragments in reverse order, but reaches a plateau for all emotions after hearing the last three to four syllables of the utterance. When viewed broadly, these findings suggest that “prototypical” acoustic properties for accessing knowledge about basic emotions from speech (Laukka, 2005; Pell et al., 2009) are decoded and consciously recognized at peak accuracy levels after processing three to four spoken syllables—approximating a mean stimulus duration of 600–1200 ms, depending on the emotion in question (review Table 1). This broad conclusion fits with observations of two previous gating studies that gated emotional utterances in syllabic units (Pell and Kotz, 2011) or in 250 ms increments (Cornew et al., 2010). However, there were notable emotion-specific recognition patterns as a function of gate duration; when stimuli were very short (i.e., only the final one or two syllables were presented) there was a marked advantage for detecting *fear* and *anger* when compared to the other expression types, and listeners were significantly more confident that they had correctly identified these two emotions based solely on the utterance-final syllable. As the gate duration gradually increased to five syllables (Gate_3-7), no further differences were observed in the ability to recognize *anger, sadness*, and *happiness*, although participants remained significantly more accurate for *fear* and significantly less accurate for *disgust* at all stimulus durations.

The observation that *fear*, and to a lesser extent *anger*, were highly salient to listeners at the end of utterances even when minimal acoustic information was present (i.e., the final syllable) is noteworthy. Leinonen and colleagues (1997) presented two-syllable emotional utterances in Finnish (the word [saara]) and reported higher recognition scores and distinct acoustic attributes of productions conveying *fear* and *anger* when compared to eight other emotional-motivational states, suggesting that these emotions are highly salient to listeners in acoustic stimuli of brief duration. Similarly, Pell and Kotz (2011) reported that recognition of most emotions improved over the *full course* of the utterance when they were gated from sentence onset and that certain emotions, such as *happiness* and *fear*, demonstrated clear gains in that study when listeners processed the last two syllables of the utterance. When combined with our current findings, this implies that syllables located towards the *end* of an utterance provide especially powerful cues for identifying basic emotions encoded in spoken language. This argument is supported by our direct statistical comparisons of the two data sets when utterances were gated from their onset vs. offset; we found that presentation *direction* had a significant impact on the accuracy and confidence levels of English listeners, with improved recognition of all emotions except *neutral* when participants heard utterances commencing with the last syllable. Gating utterances from their offset also reduced mean emotion identification times for some emotions (*happiness*, *disgust*) as elaborated below. In contrast, there was no evidence in our data that listeners were at an advantage to recognize emotional prosody when utterances were gated from their onset, with the possible exception of accuracy rates for *sadness* that were somewhat higher in the onset condition at very short gate intervals.

Why would natural, presumably biologically-specified codes for signaling emotions in the voice (e.g., Ekman, 1992; Wilson and Wharton, 2006) bear an important relationship to the temporal features of spoken language? This phenomenon, which has been highlighted at different times (Cosmides, 1983; Scherer, 1988), could be explained by the accent structure of utterances we presented for emotion recognition and by natural processes of speech production, factors which both contribute to the “socialization” or shaping of vocal emotion expressions in the context of spoken language. It is well known that the accent/phrase structure of speech, or the relative pattern of weak vs. strong syllables (or segments) in a language, can be altered when speakers experience and convey vocal emotions (Ladd, 1996). For example, speakers may increase or decrease the relative prominence of stressed syllables (through local changes in duration and pitch variation) and/or shift the location or frequency of syllables that are typically accented in a language, which may serve as an important perceptual correlate of vocal emotion expressions (Bolinger, 1972; Cosmides, 1983). Related to the notion of local prominence, there is a well-documented propensity for speakers to lengthen syllables located in word- or phrase-final position (“sentence-final lengthening,” Oller, 1973; Pell, 2001), sometimes on the penultimate syllable of certain languages (Bolinger, 1978), and other evidence that speakers modulate their pitch in final positions to encode gradient acoustic cues that refer directly to their emotional state (Pell, 2001) to give to the final position of sentences a special impact in the identification of the emotional quality of the voice.

The observation here that cues located toward the end of an utterance facilitated accurate recognition of most emotions in English likely re-asserts the importance of accent structure during vocal emotion processing (Cosmides, 1983; Ladd et al., 1985). More specifically, it implies that sentence-final syllables in many languages could act as a vehicle for reinforcing the speaker's emotion state *vis-à-vis* the listener in an unambiguous and highly differentiated manner during discourse (especially for *fear* and *anger*). Inspection of the mean syllable durations of gated stimuli presented here and by Pell and Kotz (2011) confirm that while there were natural temporal variations across emotions, the duration of utterance-final syllables (*M* = 386 ms, range = 329–481) was more than double that of utterance-initial syllables (*M* = 165 ms, range = 119–198), the latter of which were always unstressed in our study. In comparison, differences in the cumulative duration of gates composed of two syllables (*M* = 600 vs. 516 in the offset vs. onset conditions, respectively) or three syllables (*M* = 779 vs. 711) were relatively modest between the two studies, and these stimulus durations were always composed of both weak and stressed syllables. This difference of duration observed is in line with the above described propensity of speakers to lengthen syllables located in the final position of the sentences. Also, given the structure of the pseudo-utterances (see Section Appendix), it should be noted that the forward presentation of pseudo-utterances might differ from the backward presentation in terms of expectations of the participants. In Pell and Kotz (2011), the first gate was always a pronoun or a determiner and was always followed by the first syllable of a pseudo-verb, whereas in the present experiment, the two first gates were always the two final syllables of a pseudo-word. It is difficult to know whether participants may have developed some expectations about the following syllable and to what extent these expectations could have impacted the identification of the prosody. We cannot exclude that these expectations could have been more difficult to make in the backward condition, when the gates were presented in a reverse order, altering how participants focused on the emotional prosody of the sentences. However, such an interpretation would not explain why the direction of presentation did not influence the performance of participants when sentences were uttered with a neutral note and why this influence was limited to some specific gates when the sentences were spoken in an emotional way.

Nevertheless, these results suggest that there is a certain alignment in how speakers realize acoustic targets that refer to semantically-dictated stress patterns and emotional meanings in speech, demonstrating that recognition of vocal emotional expressions is shaped to some extent by differences in the temporal (accent) structure of language *and* that emotional cues are probably not equally salient throughout the speech signal. Further studies that compare our findings with data from other languages will clearly be needed to advance specific hypotheses about how vocal emotion expressions may have become “domesticated” in the context of spoken language. For example, we could replicate forward and backward gating experiments in another stressed-language like German, and see if critical cues in the identification of some emotions could be located at different places of a sentence. We could also compare forward and backward presentation of pseudo-sentences in a language that does not use accentuated stress such as French, which supposedly would lead to similar results in the time needed to identify emotional prosody irrespective of the direction of presentation of the sentences.

#### Further reflections on the time course of vocal emotion recognition

While our data show that the position of emotionally meaningful cues plays a role in how vocal emotions are revealed to listeners, they simultaneously argue that the average *time* needed to accurately decode most basic emotions in speech is relatively constant irrespective of gating method (syllables vs. 250 ms increments) or stimulus set (Cornew et al., 2010; Pell and Kotz, 2011). When mean emotion identification times were computed here, *fear* required the least amount of stimulus exposure to recognize (*M* = 427 ms), followed by *sadness* (*M* = 612 ms), *neutral* (*M* = 654 ms), *anger* (*M* = 677 ms), *happiness* (*M* = 811 ms), and *disgust* (*M* = 1197 ms). With the exception of *neutral* which took slightly (although not significantly) longer to detect when utterances were gated in reverse, this emotion-specific pattern precisely mirrors the one reported by Pell and Kotz (2011) for the same six emotions and replicates Cornew et al.'s (2010) data for *neutral*, *anger*, and *happy* expressions when utterances were gated in 250 ms units. When the mean emotion identification times recorded here are compared to those reported by Pell and Kotz (2011) and Cornew et al. (2010), it can be said that recognition of *fear* occurs approximately in the range of 425–525 ms (427, 517 ms), *sadness* in the range of 600 ms (612, 576 ms), *anger* in the range of 700 ms (677, 710, 723 ms), *happiness* in the range of 800–900 ms (811, 977, 802 ms), and *disgust* requires analysis of at least 1200 ms of speech (1197, 1486 ms). As pointed out by Pell and Kotz (2011), the time needed to identify basic emotions from their underlying acoustic cues does not simply reflect characteristic differences in articulation rate across emotions (e.g., Banse and Scherer, 1996; Pell et al., 2009), since expressions of *sadness* are routinely slower and often twice the duration of comparable *fear* expressions, and yet these two emotions are accurately recognized from speech stimuli of the shortest duration. Rather, it can be claimed that prototypical cues for understanding vocal emotions are decoded and consciously retrievable over slightly different epochs in the 400–1200 ms time window, or after hearing roughly 2–4 syllables in speech. The idea that emotional meanings begin to be differentiated after hearing around 400 ms of speech fits with recent priming data using behavioral paradigms (Pell and Skorup, 2008) and event-related potentials (ERPs, Paulmann and Pell, 2010) as well as recent neuro-cognitive models on the time course and cognitive processing structure of vocal emotion processing (Schirmer and Kotz, 2006).

Evidence that vocal expressions of certain negative emotions, such as *fear, sadness*, or *anger*, require systematically less auditory input to decode accurately, whereas expressions of *happiness* and *disgust* take much longer, may be partly explained by the evolutionary prevalence and significance of negative emotions over positive emotions (e.g., Cacioppo and Gardner, 1999). Expressions that signal threat or loss must be decoded rapidly to avoid detrimental outcomes of great urgency to the organism; this negativity bias has been observed elsewhere in response to facial (Carretié et al., 2001) and vocal expressions of fear and anger (Calder et al., 2001, 2004), and would explain why *fear* prosody was recognized more accurately and *faster* than any other emotional expression in the voice (Levitt, 1964). The biological importance of rapidly differentiating negative vocal signals (e.g., Scherer, 1986) potentially explains why the *amount* of temporal acoustic information, and not the position of critical cues, appears to be the key factor governing the time course of recognizing *fear, anger*, and *sadness*, since we found no significant differences in emotion identification times for these emotions between our two studies.

In contrast, *happy* and *disgust* took significantly longer to identify and were the only emotions for which recognition times varied significantly as a function of gating direction (with a reduction in emotion recognition times of approximately 200 ms and 300 ms between studies, respectively). Difficulties recognizing *disgust* from prosody are well documented in the literature (Scherer, 1986; Scherer et al., 1991; Jaywant and Pell, 2012) and are sometimes attributed to the fact that *disgust* in the auditory modality is more typical in the form of affective bursts such as “yuck” or “eeeew” (Scherer, 1988; Simon-Thomas et al., 2009). It is possible that identifying disgust from running speech, as required here and by Pell and Kotz (2011), activates additional social meanings that take more time to analyze and infer than the decoding of pure biological signals such as *fear, sadness*, and *anger*. For example, it has been suggested that there are qualitatively different expressions of disgust in the visual (Rozin et al., 1994) and auditory (Calder et al., 2010) modality, including a variant related to violations of moral standards that is often conveyed in running speech, as opposed to physical/visceral expressions of disgust which are better conveyed through exclamations (yuck!). If presentation of disgust utterances engendered processes for inferring a speaker's social or moral attitude from vocal cues, a more symbolic function of prosody, one might expect a much slower time course as witnessed here. A similar argument may apply to our results for *happiness*; although this emotion is typically the quickest emotion to be recognized in the visual modality (Tracy and Robins, 2008; Palermo and Coltheart, 2004; Calvo and Nummenmaa, 2009), it exhibits a systematically slower time course in spoken language (Cornew et al., 2010; Pell and Kotz, 2011). Like disgust, *happiness* may also be communicated in a more rapid and reliable manner by other types of vocal cues that accompany speech, such as laughter (e.g., Szameitat et al., 2010). In addition, there is probably a need to differentiate between different types of vocal expressions of happiness which yield different rates of perceptual recognition (Sauter and Scott, 2007). Nonetheless, our results strongly imply that speakers use prosody to signal *happiness*, particularly towards the end of an utterance, as a conventionalized social cue directed to the listener for communicating this emotion state (Pell, 2001; Pell and Kotz, 2011), perhaps as a form of self-presentation and inter-personal expression of social affiliation. Further inquiry will be needed to test why *disgust* and *happiness* appear to be more socially mediated than other basic emotions, influencing the time course of their recognition in speech, and to define the *contexts* that produce variations in these expressions.

Interestingly, the recognition of *neutral* prosody was uniquely unaffected by the manner in which acoustic information was unveiled in the utterance, with no significant effects of presentation direction on accuracy, confidence ratings, or emotion identification times between studies. This tentatively suggests that the identification of neutrality, or a lack of emotionality in the voice, can be reliably inferred following a relatively standard amount of time in the range of 400–650 ms of stimulus exposure (Cornew et al., 2010; Pell and Kotz, 2011). Since our measures of recognition include conscious interpretative (naming) processes and are biased somewhat by the gating method, our data on the time course for *neutral* prosody are not inconsistent with results showing the *on-line* differentiation of neutrality/emotionality in the voice at around 200 ms after speech onset, as inferred from amplitude differences in the P200 ERP component when German utterances were presented to listeners (Paulmann et al., 2008). One can speculate that listeners use a heuristic or default process for recognizing *neutral* voices whenever on-line analysis of prosody does not uncover evidence of emotionally meaningful cue configurations; presumably, this process for rejecting the presence of known acoustic patterns referring to emotions, like the process for decoding known patterns, is accomplished over a relatively stable time interval. To test these possibilities, it would be interesting to modify neutral sentences by inserting local variations in emotionally-meaningful acoustic features at critical junctures in time to determine if this “resets the clock” for inferring the presence or absence of emotion in speech.

## Conclusion

Following recent on-line (ERP) studies demonstrating that vocal emotions are distinguished from neutral voices after 200 ms of speech processing (Paulmann and Kotz, 2008), and that emotion-specific differences begin to be detected in the 200–400 ms time window (Alter et al., 2003; Paulmann and Pell, 2010), our data shed critical light on the time interval where different emotion-specific meanings of vocal expressions are fully recognized and available for conscious retrieval. While it seems likely that the phrase structure of language governs local opportunities for speakers to encode emotionally-meaningful cues that are highly salient to the listener, at least in certain contexts, there are remarkable consistencies in the *amount* of time listeners must monitor vocal cue configurations to decode emotional (particularly threatening) meanings. As such, the idea that there are systematic differences in the time course for arriving at vocal emotional meanings is confirmed. To gather further information on how social factors influence the communication of vocal emotional meanings, future studies using the gating paradigm could present emotional utterances to listeners in their native vs. a foreign language; this could reveal whether specificities in the time course for recognizing emotions manifest in a similar way for native speakers of different languages, while testing the hypothesis that accurate decoding of vocal emotions in a foreign language is systematically delayed due to interference at the phonological level (Van Bezooijen et al., 1983; Pell and Skorup, 2008).

### Conflict of interest statement

The authors declare that the research was conducted in the absence of any commercial or financial relationships that could be construed as a potential conflict of interest.

## Appendix

A list of pseudo-utterances produced to convey each target emotion that were gated for presentation in the experiment.

1. I tropped for swinty gowers.
2. She kuvelled the noralind.
3. The placter jabored the tozz.
4. The moger is chalestic.
5. The rivix joled the silling.
6. The crinklet is boritate.
7. She krayed a jad ralition.
8. We wanced on the nonitor.
9. They pannifered the moser.
10. We groffed for vappy laurits.
11. I marlipped the tovity.
12. The varmalit was raffid.
13. They rilted the prubition.
